# Engineering Crystal Packing in RNA Structures I: Past and Future Strategies for Engineering RNA Packing in Crystals

**DOI:** 10.3390/cryst11080952

**Published:** 2021-08-15

**Authors:** Narsimha Pujari, Stephanie L. Saundh, Francis A. Acquah, Blaine H. M. Mooers, Adrian R. Ferré-D’Amaré, Adelaine Kwun-Wai Leung

**Affiliations:** 1 Department of Veterinary Biomedical Sciences, University of Saskatchewan, Saskatoon, SK S7N 5B4, Canada; 2 Department of Biochemistry and Molecular Biology, University of Oklahoma Health Sciences Center, Oklahoma City, OK 73104, USA; 3 Stephenson Cancer Center, University of Oklahoma Health Sciences Center, Oklahoma City, OK 73104, USA; 4 Biochemistry and Biophysics Center, National Heart, Lung and Blood Institute, Bethesda, MD 20892, USA

**Keywords:** ribonucleic acid, RNA, X-ray crystallography, crystal packing design, RNA motifs, nucleic acid crystallography, RNA crystallization constructs, RNA crystallography, RNA crystallogenesis

## Abstract

X-ray crystallography remains a powerful method to gain atomistic insights into the catalytic and regulatory functions of RNA molecules. However, the technique requires the preparation of diffraction-quality crystals. This is often a resource- and time-consuming venture because RNA crystallization is hindered by the conformational heterogeneity of RNA, as well as the limited opportunities for stereospecific intermolecular interactions between RNA molecules. The limited success at crystallization explains in part the smaller number of RNA-only structures in the Protein Data Bank. Several approaches have been developed to aid the formation of well-ordered RNA crystals. The majority of these are construct-engineering techniques that aim to introduce crystal contacts to favor the formation of well-diffracting crystals. A typical example is the insertion of tetraloop–tetraloop receptor pairs into non-essential RNA segments to promote intermolecular association. Other methods of promoting crystallization involve chaperones and crystallization-friendly molecules that increase RNA stability and improve crystal packing. In this review, we discuss the various techniques that have been successfully used to facilitate crystal packing of RNA molecules, recent advances in construct engineering, and directions for future research in this vital aspect of RNA crystallography.

## 1. Introduction

Structural biology of RNA molecules began in the 1960s with the stepwise resolution improvement of the yeast tRNA^Phe^ crystal structures [1–3]. The cloverleaf structure of the tRNA is likely the first introduction to the RNA structure encountered by many science students. The tRNA crystal structures unveiled how specific tertiary interactions maintain the three-dimensional fold of the RNA molecule, and how the three-dimensional architecture enables its role in protein translation. The next breakthrough came in the crystal structures of the ribozymes [4–6] and self-splicing introns [7], which demonstrated how RNA can fold into an architecture capable of carrying out enzymatic catalysis. These early RNA crystal structures set the stage for the structural investigation of large RNA-containing macromolecular complexes such as the ribosome and the spliceosome that are key players in the central dogma of molecular biology [8–10]. The millennium began with the discovery of many more types of non-coding RNAs (e.g., microRNAs, riboswitches, lncRNAs; see [11] for a comprehensive review). X-ray crystallography has remained a critical experimental approach to understanding the molecular mechanisms enabling these non-coding RNAs to perform extraordinarily diverse biological functions.

Crystallization of RNA molecules for structural determination and molecular interaction elucidation is often more challenging than the crystallization of soluble proteins [12,13]. This in part explains why RNA-only structures account for less than 0.7% of the biomolecular crystal structures deposited in the Protein Data Bank (PDB). As an equilibrium process, crystallization is governed by both the properties of the soluble molecule and the nature of its crystalline state. Although RNA duplexes are highly stable thermodynamically, the higher-order folding landscape of RNA is often complex, with multiple competing minima, as well as kinetic traps [14–17]. This results in conformational heterogeneity in the solution [18]. The molecular surface of folded RNAs is dominated by a regular array of negatively charged phosphates that can lead to packing (through counterions) into crystals that are disordered at the atomic level [19–21]. Strategies for improving conformational homogeneity of folded RNAs are generally useful for any biophysical or structural study of this biomolecule, while techniques for enhancing the formation of improved crystal contacts are often necessary for the successful application of X-ray, neutron, or electron crystallography [12,22–26].

The idea of engineering nucleic acid sequence to promote crystal contacts originated in the work of Schultz et al., where they successfully crystallized the *E. coli* catabolite gene activator protein complexed with its DNA-binding site after scanning through 26 different DNA sequences [27]. Over the years, several molecular engineering techniques have been developed to favor ordered crystal packing of RNAs (e.g., [28–30]). Furthermore, optimized crystallization conditions and sometimes post-crystallization treatments have also been crucial for success in RNA crystallography [31,32]. In this review, we highlight new engineering strategies for RNA crystallization, as well as techniques that have proved successful in aiding the growth of diffraction quality crystals in the past [33]. In addition, we emphasize considerations that may affect their adoption and use in different RNA contexts beyond where they were initially developed [34]. This review may be of interest to nucleic acid crystallographers who seek to expand their repertoire of engineering tools to tackle the crystallization of challenging RNA targets.

## 2. The Propensity of RNA Helices to Form Intermolecular Stacks

The simplest RNA interactions are formed by Watson-Crick (WC) base paired duplexes. The duplex can form in *cis* between adjacent complementary sequences generating a loop structure or between two complementary strands far away in the primary sequence but brought together through more complicated intermediate RNA structures. Duplexes can also form between two RNA strands binding in *trans*, which is common in trypanosome RNA editing, RNA viruses, and RNA interference. The stacking of terminal base pairs between RNA helices is a common packing scheme observed in many crystals. Therefore, engineering helical ends to encourage inter-helical stacking is an excellent strategy to promote or improve crystal packing (for historical context, see, e.g., [35]). The length of the duplex may be the primary determinant of the angle between the long axes of the terminal base pairs at the junction between two stacked duplexes. This angle can be positive or negative, and the helical axes of the duplexes can be aligned or offset. When the axes are offset, the ribose ring of one terminal nucleotide may pack against the terminal base pair of the adjacent duplex and be correlated with buckling of the terminal base pair. AU base pairs may tolerate this buckling better than GC base pairs, so the identity of the terminal base pairs and chain length may be important determinants of helical stacking. Note that the effects of AU and UA base pairs in the terminal positions are probably not equivalent. The inclusion of terminal phosphates [30] and overhanging nucleotides can also influence success at crystallization. An example of the latter was illustrated in structural studies of the minimal hairpin ribozyme [25]. Packing analysis of a crystal that diffracted to 3.17 Å resolution revealed suboptimal intermolecular helical stacking at the crystal packing interface. The RNA construct was subsequently re-designed to have a single U “sticky” end that permitted a more optimal helical stacking and crystalline order, thereby increasing the diffraction limit to 2.05 Å.

There are three notes of caution when designing constructs to study RNA duplexes. First, the stability of duplex stacking can often lead to the crystallization of duplexes other than those intended. This result is most problematic when other conformations of RNA, such as stem-loops, are the subject of study, but crystallization selects non-desirable duplex forms [36,37]. Second, when duplexes are formed from two different RNA strands, the heteroduplexes are prone to an end-on-end disorder that can promote crystal twinning [20]. Because the phosphate backbone is symmetrical while the base pairings are not, the helices do not have to be packed consistently in the same orientation. A twinned data set will occur if a fraction of the helices is flipped while maintaining the same inter-helical packing. To avoid this crystal packing artifact, it is advisable to fuse two copies of the duplex as has been done successfully for fragments of the U-tail:pre-mRNA duplex from a trypanosome RNA editing substrate [38,39]. The substrates were 8- and 16-base pairs long and formed duplexes with 16- and 32-base pairs, respectively. The longer construct actually gave 1.05 Å diffraction data suitable for ab initio structure determination by direct methods despite the presence of translational pseudosymmetry due to the three helical turns in the RNA duplex [40]. Third, because crystallization is only compatible with 1-, 2-, 3-, 4-, and 6-fold rotations [41], when there are duplexes whose helical pitches are not compatible with this requirement (i.e., their length does not correspond to a multiple of a full, 1/2, 1/3, 1/4, or 1/6th helical turn) crystals can form where the molecule is incommensurate with the crystallographic unit cell. An example is the crystallization of an 18-mer A-form duplex that resulted in the RNA packing in 36 different registers; any of its residues occupied any one helical position in the crystal. That is, the bases in the structure were disordered 36-fold, while the sugar-phosphate backbone of the RNA adopted apparently identical (at 1.2 Å-resolution) conformations at all positions [19]. This kind of disorder, a special form of twinning, is readily apparent in the distribution of scattering factor intensities [21].

It is not easy to predict accurately whether a short RNA will crystallize as a hairpin or a duplex. Many of the early crystal structures of RNA duplexes about one helical turn in length had mismatch bases in the middle with flanking Watson–Crick base pairs, for example, the RNA dodecamer duplex (r-GGACUUCGGUCC)2 [42]. These RNA duplexes were failed attempts at crystallizing RNA hairpins with tetraloops. The high concentration of RNA in the crystallization drop and the stable crystalline packing of the duplex drove disproportionation. The undesired duplex formation was favored in the crystallization of a 27-nt RNA that was crystallized under solution conditions that favored hairpin formation [43]. The conformation of single-stranded RNAs is easily checked by native PAGE with the appropriate control RNA species of known duplex or hairpin conformation, but the oligomerization state of an RNA in solution may differ from that favored by crystallization [44,45].

## 3. Hairpin Loops and Their Utility in Crystal Packing Design

When adjacent complementary sequences hybridize to form an RNA duplex, a hairpin or stem loop structure is formed. Proteins can bind some loops (e.g., spliceosomal proteins U1A [46], U2A’/B” [47], the pseudouridine synthase TruB [48]). Other loops can serve as folding nucleation sites, confer stability, and participate in tertiary RNA interactions (e.g., the UUCG tetraloop [49]). Tetraloops and kissing loops are the most common hairpin loops that engage in tertiary RNA interactions. Replacing flexible structures between complementary sequences by tetraloops or kissing loops is a common strategy to promote the formation of well-packed crystals by removing undesirable elements that hinder crystallization and generating potential interfaces for crystal packing. We review several specific examples below to illustrate these principles in action.

### 3.1. Promoting Loop-Loop Crystal Contacts: The Kissing Loop Complex

**Figure 1.**
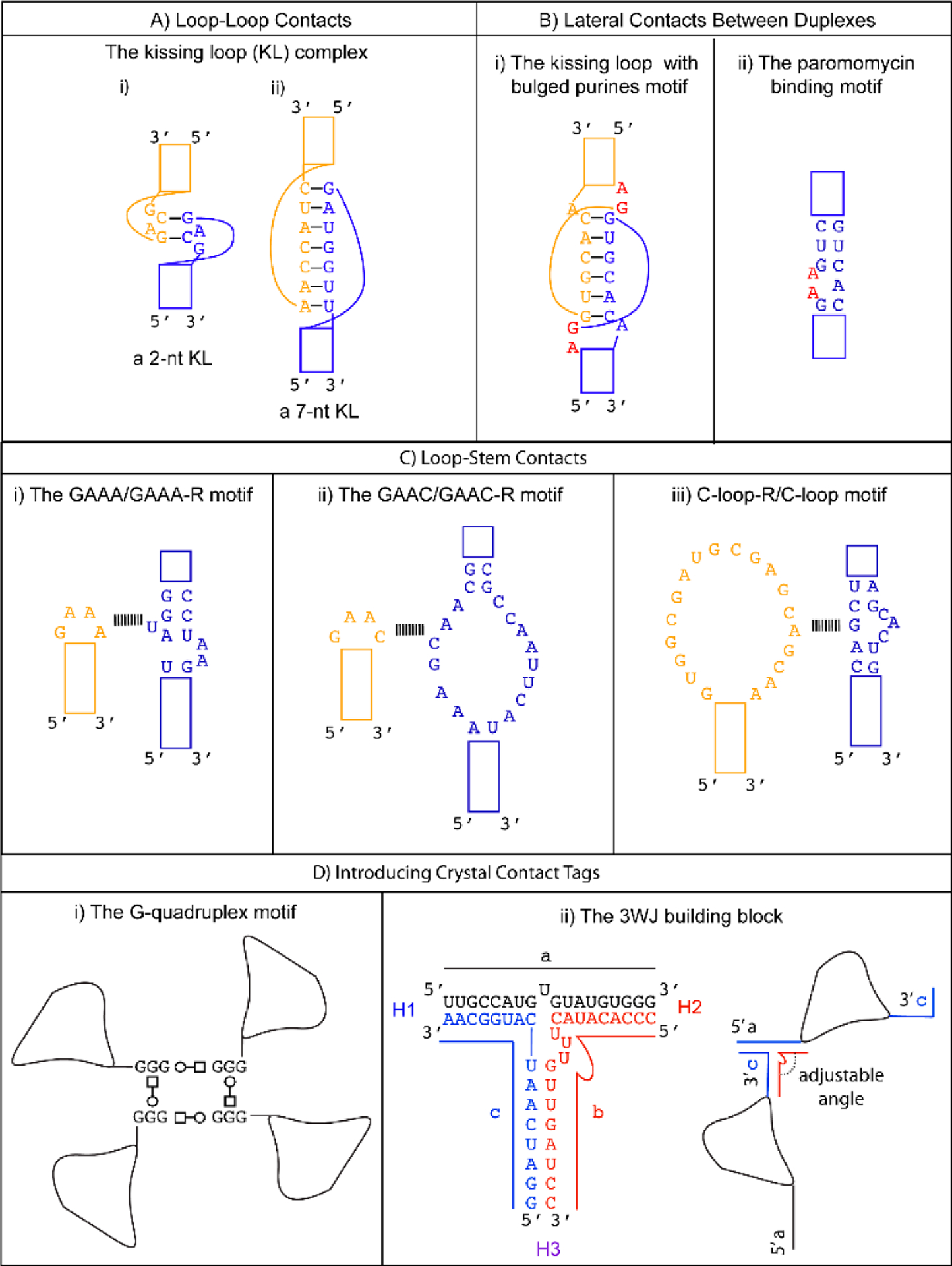
Summary of motifs discussed in the review. (**A**) Two examples of the kissing loop (KL) complex. (**i**) A 2-nt KL complex is formed between the H3 stem loop of the Moloney Murine leukemia virus [54]. (**ii**) A 7-nt KL complex is formed between stem loops I and II of the ColE1 plasmid [55,56]. (**B**) Design options to induce lateral contacts between duplexes. (**i**) A kissing loop complex with flipped-out purines from the dimerization initiation site of the HIV-1 genome [60]. (**ii**) The paramomycin binding site from the 16S rRNA of the *E. coli* ribosome can be utilized to introduce bulged adenines that can form tertiary interactions with a neighboring backbone [61,62]. (**C**) Design options to induce loop to stem contacts. (**i**) The classical GNRA tetraloop (GAAA) interacting with its 11-nt receptor GAAA-R. (**ii**) The non-GNRA tetraloop (GAAC) interacting with its 20-nt receptor GAAC-R identified by in vitro selection. (**iii**) The asymmetrical C-loop interacting with its 20-nt receptor C-loop-R identified by in vitro selection. (**D**) Design option to introduce a crystal contact tag. (**i**) A G-rich tag can be introduced to the ends of a nucleic acid structure to induce the formation of a G-quadruplex. The squares and circles indicate the tertiary interactions following the Leontis and Westhof nomenclature [63]. A circle indicates a Watson–Crick edge, and a square indicates a Hoogsteen edge. Open or white color indicates *trans* orientation of the glycosidic bond. (**ii**) The three-way junction motif (3WJ) can be self-assembled from three oligonucleotides (strands a, b, c). One way to utilize this motif is to engineer strands a and c into the ends of the RNA. The third strand b can be supplied in the crystallization drop. If a crystal is formed, the packing geometry can be fine-tuned by simply changing strand b with a different number of unpaired nucleotides at the junction.

The kissing loop is an RNA tertiary interaction motif that can be used for designing sequence-specific loop-loop contacts. Found in natural RNAs, the kissing loop formation is essential for the regulation of the ColE1 plasmid replication [50], and the dimerization of the viral genome that occurs during the life cycle of HIV-1 [51] and the Hepatitis C virus [52]. The kissing loop complex is formed by Watson–Crick base pairing between the complementary palindromic sequences of the two unpaired stem loops. The first kissing loop complex was visualized in the crystal structure of the yeast tRNA^Asp^ where its anti-codon forms a 3-nt kissing loop complex with the anti-codon of a symmetry-related mate [53]. Since then, kissing loops with variable lengths have been observed in other structures. For example, the H3 stem loop of the Moloney murine leukemia virus forms a homodimeric kissing loop complex with just two intermolecular GC base pairs [54] (Figure 1A-i). On the other hand, the RNA I/II kissing loop complex from the ColE1 plasmid is formed between 7-nts from the two RNA stem loops [55,56] (Figure 1A-ii). The versatility of the kissing loop tertiary motif was exploited in the crystallization of the spliceosomal U1 snRNP [57–59].

### 3.2. Promoting Loop to Stem Crystal Contacts: Loop to Receptor Motifs

#### 3.2.1. Tetraloop-Tetraloop Receptor Motifs

A tetraloop is a 4-nt loop that caps a stem. The GNRA and the UNCG (N = any nucleotides, R = either purine) families are the most common types found in natural RNAs. The GNRA tetraloop typically adopts a U-turn conformation, characterized by the first nucleobase forming a hydrogen bond with the backbone of the fourth nucleotide. This base to backbone interaction positions the first nucleobase to form a stacking (π-OP) interaction with the oxygen of the phosphate backbone of the third nucleotide. The UNCG tetraloop typically adopts a Z-turn conformation, characterized by the first and fourth nucleotides forming a *trans* Sugar/WC interaction and an unusual ribose backbone conformation that allows the formation of a stacking (π-O4′) interaction between the fourth nucleobase and the ribose oxygen of the third nucleotide [64]. The GNRA tetraloops are more commonly found to engage in tertiary interactions. In contrast, the UNCG tetraloop has exceptional thermostability, and it is typically used to replace flexible regions undesirable for crystal packing. The thermal stability of the UNCG tetraloop is mainly contributed by hydrogen bonds between the 2′-hydroxyl groups of the first and second sugar moiety with the Hoogsteen edge of the base of the fourth nucleotide [65,66]. Interestingly, the core of the four-way junction is recently identified as a receptor for a UNCG tetraloop [67]. However, a four-way junction is not easy to implement in other structural contexts. We will consider tetraloops with their cognate receptors that are convenient to incorporate in different RNA structures to promote loop to stem contacts.

##### The GAAA Loop and Its 11-nts Receptors (GAAA-R)

One of the most common GNRA tetraloops, the GAAA tetraloop, was first observed to interact with the ribose moiety in the minor groove of an A-form helix without much sequence specificity [5,68]. However, its long-range tertiary interaction with a sequence-specific region on the self-splicing group I intron was deduced and characterized by chemical probing [69] and mutagenesis [70]. The 11-nt sequence motif was identified as the GAAA receptor (hereafter, GAAA-R) by co-variation analysis [71]. The detailed interaction between the GAAA tetraloop and the 11-nt receptor (GAAA-R) was finally revealed in the crystal structure of the P4-P6 domain of the group I self-splicing intron [7]. In vitro selection of different types of GNRA tetraloops has identified other receptor sequences, showcasing the versatility of the tetraloop/receptor motif [72,73]. The GAAA-R motif can be engineered anywhere on an RNA helix, thus providing a loop to duplex interaction design option (Figure 1C-i). The GAAA/GAAA-R interaction is used in a popular strategy [29] to promote RNA crystal formation and has found numerous applications, for instance, to promote crystallization of the spliceosomal U4 snRNP core domain [59,74,75]. Crystallization of the Ribonuclease P holoenzyme in complex with tRNA^Phe^ was achieved by introducing the GAAA tetraloop into the RNase P and the GAAA-R to the tRNA [76]. More recently, the GAAA and GAAA-R were engineered to facilitate the crystallization of a short RNA helix with CUG repeats, which occur in myotonic dystrophy when CTG in the 3′ UTR of a gene is abnormally expanded. The transcribed CUG repeats can form a stable A-form RNA duplex that sequesters RNA binding proteins and thereby interferes with the normal functions of the sequestered proteins. The incorporation of GAAA/GAAA-R into the CUG repeat duplex has an added advantage in that it created a large solvent-accessible space within the crystal. As a result, the crystal form could become a convenient platform to screen potential molecules that can target the CUG repeat duplex for diagnostic or therapeutic purposes [28].

##### The GAAC Loop and Its 20-nts Receptor (GAAC-R)

The GAAC is a non-GNRA tetraloop found in group II introns and has a folding geometry distinct from the GNRA fold [77,78]. Instead of a *trans* Sugar/Hoogsteen base pair between the first and fourth nucleotide, a *trans* Sugar/WC base pair was observed. As a result, the first base of the loop no longer stacks with the 5′ WC base pair of the stem. A modular 20-nt receptor was identified by in vitro selection with a binding affinity (kD ~ 2.4 nM) comparable to that of the GAAA/GAAA-R interaction [79] (Figure 1C-ii). The GAAC/GAAC-R loop to stem interaction may have a different orientation compared to that formed by GAAA/GAAA-R. This is based on the relative catalytic efficiency observed in the in vitro selection system. The tetraloop and its receptor are engineered in a ribozyme structural fold, and the ribozyme catalysis depends on their interaction. Although the GAAC/GAAC-R motif resulted in catalysis, the efficiency was ~30% less than that observed for the GAAA/GAAA-R motif [80]. The authors attributed the catalytic difference to a variation in the orientation of the interaction motif. Thus, the GAAC/GAAC-R motif could create alternative packing geometry compared to the GAAA/GAAA-R motif. Unfortunately, no structural confirmation of the interaction is available to date. Nonetheless, a chemical probing experiment suggests that the WC edges of the middle two As interact with the receptor [79].

#### 3.2.2. The C-Loop and Its 20-nts Receptor (C-loop-R)

The C-loop is a recurrent motif characterized by an asymmetric internal loop. As observed in ribosome structures, the main structural function of the C-loop is to increase the helical twist of the stem loop where it is embedded so that the hairpin loop can engage in optimal tertiary interaction [80]. Although it does not naturally participate in tertiary interaction, a 20-nts loop receptor (C-loop-R) in the form of a loop was identified by in vitro selection [81] (Figure 1C-iii). Therefore, a possible crystal packing design incorporates a C-loop motif into a stem, and the C-loop-R could be inserted as a stem loop. Unfortunately, no structural information of the interaction is available. However, chemical probing experiments suggest that the C-loop motif binds C-loop-R via non-WC interactions [81].

## 4. Designing Lateral Contacts between Duplexes

Terminal base pair stacking of intermolecular duplexes and loop-loop interaction of the kissing loops will promote crystal contact along the same direction of the helical axis. Lateral packing of helices frequently involves ribose zipper motifs that utilize two consecutive base pairs from two regions of the RNA stem running in an anti-parallel fashion. Different types of ribose zippers are characterized by their base-backbone and base-base interaction patterns in the minor grooves [82,83]. Although there is a certain degree of sequence specificity for the ribose zipper, its variability makes it difficult to use as a general motif for crystal packing design. In this section, we will discuss several design options that can promote lateral interaction of RNA helices.

### 4.1. Kissing Loop with Two Bulged Purines

In addition to the typical kissing loop complex introduced earlier, an unusual kissing loop complex with two bulged purines was observed in an RNA duplex fragment that contains the HIV-1 genome dimerization initiation site (DIS). The DIS contains a 9-nt loop with two conserved purines at the 5′ end, followed by a 6-nt sequence that makes kissing loop contacts and an invariant adenine at the 3′ end (Figure 1B-i). The crystal structure of the DIS complex contains a 7-bp WC stem capped by the DIS loop. As expected, the two hairpins form a head-to-head pseudo-continuous coaxial A-form duplex via WC base pairing of the 6-nt complementary sequence. Interestingly, the two conserved purines 5′ to the kissing sequence are flipped out to form crystal contacts lateral to the duplex by stacking with their symmetry-equivalent mates, while the 3′ conserved adenine remains unpaired within the helix [60]. While the biological relevance of this unusual kissing complex is controversial [84,85], the same bulged out residues were observed in three different sequences, crystallization conditions, and space groups [60]. Therefore, this is likely a recurrent motif that could be useful if a coaxial duplex with lateral stabilization is desired in the crystal packing design. This kissing loop with two bulged purines was utilized to improve the diffraction quality of the U1 snRNP crystal [57,59].

### 4.2. Paromomycin Binding Motif

Another motif that can be used to stabilize duplexes laterally is the paromomycin motif. Paromomycin is an antibiotic that binds to the A site of the 16S rRNA of the *E. coli* ribosome. Its interaction causes structural changes that render the ribosome unable to discriminate near-cognate tRNA [86]. The crystal structure of an 18-bp helix with two 13-nt paromomycin binding sites incorporated between WC base-pairs shows that the binding of paromomycin flips out two adenines, which then form critical A-minor motif interactions with the neighboring backbones [61,62] (Figure 1B-ii). In an attempt to improve the diffraction quality of the spliceosomal U4 snRNP core domain, the paromomycin motif was incorporated into the U4 snRNA. Indeed, crystals in different space groups were obtained only in the presence of paromomycin [59,74]. However, the diffraction limit did not improve, highlighting the reality that improving packing geometry is much more difficult than promoting crystal formation.

## 5. Introducing Crystal Contact Tags

The ends of the RNA could be another accessible point for engineering crystal packing motifs. Here we will suggest two possibilities: the G-quadruplex and the three-way junction module.

### 5.1. G-Quadruplex

The G-quadruplex is a natural structure found in G-rich regions of nucleic acids (e.g., telomeric DNA and RNA). The quartet adopts a square and planar structure where four guanine bases form hydrogen-bonding interactions through their WC and Hoogsteen edges (the structures of RNA G-quadruplexes have been reviewed recently [87]). In the crystal structure of the σ-subunit of RNA polymerase in complex with a 10-nt ssDNA, the 3′ terminal GGG flips out into the solvent to form a pseudo-continuous G-quadruplex column, which forms the main crystal lattice [88]. At the crystal packing interface, a string of 3 G’s flipped out from each complex to form three sets of *trans* Hoogsteen/WC base pairs with one symmetry-related complex and *trans* WC/Hoogsteen base pairs with another symmetry-related complex. In theory, a short single-stranded GGG RNA extension engineered at either end of the RNA structure could form a similar G-quadruplex crystal contact, potentially generating a four-fold symmetry (Figure 1D-i).

### 5.2. The Three-Way Junction (3WJ) Building Block

Recurrent RNA 3D motifs observed in RNA crystal structures have been utilized in RNA nanotechnology to build self-assembling platforms for various biomedical applications [89–92]. The self-assembly of RNA nanoparticles requires building blocks that can form long-range interactions, which is the same prerequisite as RNA modules that promote crystallization. The field of RNA nanotechnology had been exploiting RNA tertiary binding motifs that occur in natural RNAs to develop interesting ways to form symmetrical polygons known as origami [93]. These polygons can be engineered to the ends of the RNA to promote crystal packing, similar to what was envisioned by the pioneer of DNA nanotechnology, Nadrian Seeman, who first began to rationally design 3D DNA crystals with self-assembling oligonucleotides [94].

The three-way junction (3WJ) observed in biological RNAs has been developed and utilized as a versatile building block in RNA nanotechnology. The 3WJ connects three WC base-paired helices with three 1–3 nts single-stranded segments. Interestingly, the 3WJ motif can be assembled readily from three short RNA oligonucleotides in water at room temperature and the resulting complex is stable in 8 M urea (Figure 1D-ii) [95]. The lengths of the H1, H2, and H3 helices can be shortened to 6, 8, and 6 base pairs without affecting complex formation. The ability to self-assemble into a stable complex from short oligonucleotides makes this an attractive motif for engineering crystal contacts of the RNA structure. One possibility is to engineer the two strands (a (18nt) and c (16 nt)) into the 5′ and 3′ tail of the RNA structure, and the third strand (b (20nt)) can be added before crystallization. Thus, the tails of the RNA can contact each other in a head-to-tail manner, and the synthetic oligonucleotide can be used for fine-tuning the packing geometry. Because the orientation of the helices joined by 3WJ is dictated by the number of unpaired nucleotides on strand b, the packing geometry could be adjusted by changing the number of nts on the single-stranded segment of the synthetic oligonucleotide rather than reengineering the whole RNA construct [33].

## 6. Introducing RNA Binding Proteins

The molecular surface of RNA is dominated by repetitive anionic backbone phosphates. The limited chemical diversity and charge repulsion reduce the chances that the RNA molecule can pack in a unique and consistent manner. RNA also tends to be more conformationally heterogeneous. These factors impede the crystallizability of RNAs compared to proteins. To promote favorable crystal packing of RNAs, one strategy is to introduce peptides or proteins to bridge the RNA molecules.

### 6.1. RNA Binding Protein—U1A

The spliceosomal protein U1A is the first and most widely used protein module for crystallizing RNAs. The N-terminal 98-residue RNA recognition motif of U1A (U1A-RRM) recognizes stem loop II of the U1 snRNA. The 1.9 Å crystal structure of U1A bound to a fragment of stem loop II of U1 snRNA shows how the U1A-RRM interacts with the 10 unpaired nucleotides (AUUGCACUCC) on the stem loop [46]. By engineering the 10-nt U1A binding loop onto a functionally dispensable RNA stem and incorporating the mutant U1A-RBD previously engineered to improve the surface properties for crystal packing, a multitude of RNA structures have been determined. A detailed review of successful cases using the U1A module has been documented previously [96]. More recently, the U1A module was used to promote crystal packing of a fusion construct of U170K bound to the stem loop I of U1 snRNA. Like U1A, U170K also has an RRM, but it uses a different binding mechanism to interact with its stem loop [57,59].

### 6.2. Antibody Fragment

Fragments of antibody (Fabs) selected to bind RNA by phage display have been utilized to create an interface for crystal contacts. Compared to U1A, Fabs are more efficient at creating crystal contacts as they are larger (50 kDa compared to 11 kDa) and thus have more surface area available for potential crystal contacts [97]. In addition, the β-sheets in antibody structure are predisposed to make good crystal contacts [98]. Fab-assisted crystallization was successfully applied to the crystal structure of the P4-P6 domain of the Tetrahymena group I intron [99] and the artificial ribozyme, the class I ligase [97]. Interestingly, the latter structure revealed that the stem loop sequence GAAACAC is the minimal antigen for the Fab fragment developed in the study [97]. Thus, this loop sequence and its Fab fragment can be exploited as a general crystallization tag for any RNA structure. The loop can be engineered on top of any A-form helix, and crystallization can be attempted with the same antibody fragment, which can be expressed and purified efficiently from *E. coli* culture [97]. In addition to promoting crystal packing, the Fab fragment can facilitate phase determination by molecular replacement and Cryo-EM map interpretation.

### 6.3. Peptide Nucleic Acid

If introducing a large artificial protein is undesirable, one could consider utilizing a synthetic molecule to form specific WC-like base pairs with the RNA. Peptide nucleic acid (PNA) is a synthetic molecule comprising of repeating N-(2-aminoethyl) glycine units, essentially resembling a peptide backbone attached to nucleobases [100]. PNA was developed into potential therapies that target abnormal nucleic acids. However, because PNA has an overall neutral charge that eliminates the intrinsic electrostatic repulsion of the phosphate backbone, it could facilitate the crystallization of RNA molecules. PNA synthesized with complementary bases can hybridize to an RNA to form a PNA/RNA duplex with slight geometric deviation from an RNA A-form helix [101]. The PNA backbone can be further modified to enhance rigidity and incorporate other functional groups [102]. Although PNA has not been utilized as a crystallization tool yet, it may be worth exploring in parallel to the crystal contact tag strategy suggested earlier.

## 7. Post-Crystallization Treatment

When incorporating interacting modules to promote crystal packing, it is important to try to place them along with several WC bp insertions to fine-tune the orientation. Each bp insertion will add a rotation of 32.7° and a rise of 2.6 Å along the helical axis [103]. If a crystal is obtained but diffraction is poor, it is vital to not give up on the construct before sufficient optimization is performed. First, the crystal needs to be of sufficient size. For the ~100 kDa U4 snRNP core domain crystal, the best diffraction data (~3.6 Å) were not obtained unless the crystal was at least 300–400 μm in size [74]. Also, it is important to perform diffraction tests at room temperature to ensure that the cryoprotection protocol is not damaging the crystal [104]. As with protein crystals [105], controlled dehydration of RNA crystals can improve diffraction limits (e.g., dehydration of *glmS* ribozyme-riboswitch crystals improved diffraction from ~3.0 Å to 1.7 Å) [106]. Finally, cation replacement coupled with dehydration should be attempted to promote repacking of the RNA [31]. Replacing 20 mM MgCl_2_ with 40 mM SrCl_2_ in the presence of a high concentration of precipitant successfully improved the diffraction limit from ~8 to ~3 Å of the crystals of the ternary complex of the Stem I domain of a T-box riboswitch, its cognate tRNA, and the RNA-binding protein YbxF [107]. Structural analysis showed that the more flexibly coordinated Sr^+^ metal cations allowed the RNA components to shift as rigid bodies and repack more optimally, thereby contributing to the remarkable improvement in diffraction quality [31]. Barium has a high coordination number like strontium and may be a suitable alternative. Finally, if all else fails, determining a low to medium resolution structure to understand the packing could inspire new designs to improve crystal packing.

## 8. Future Directions of RNA Crystallography

When prior knowledge crystallization modules and rationale design approaches fail, a stochastic process might succeed. One example is the exciting “in crystallo” selection in which error-prone PCR generates a pool of 10 million mutant DNA templates (each DNA containing 0–2 mutants). Next, the templates are transcribed with Phage T7 RNA polymerase, gel purified, and then used in crystallization experiments using conditions known to give crystals of the wild-type RNA [13]. The largest crystals are isolated, dissolved, reverse transcribed, and sequenced. In principle, the selection process is repeated for several cycles to obtain better crystals. When the second round of selection was applied to the P4-P6 domain of Tetrahymena, smaller crystals were obtained, so the selection process was abandoned. The P4-P6 domain crystallizes with two molecules in the asymmetric unit. In the first cycle, wild-type molecules were present; they may have acted as crystallization chaperones for the mutant molecules because a wild-type molecule paired with a mutant molecule in the asymmetric unit. The mutant RNAs were made and crystallized individually, and structures were determined from four of the mutants. Some of the mutant RNAs gave inferior diffraction in the absence of the wild-type RNA. The authors suggest that the selection process may be more stringent in selecting mutants that give crystals when only one RNA molecule can occupy the asymmetric unit. The authors did not explore the combination of two or more mutations to determine if the multi-mutant RNA could give crystals that diffract to a higher resolution than the wild-type RNA. The authors found improvement in the electron density maps around the mutant sites compared to the wild-type structure by local structural rearrangements in loops and new intermolecular contacts [13]. Their results suggested that bulged residues could be walked along the chain in either direction without disrupting the core structure. They also suggest that “bulge engineering” could be applied to any unpaired surface regions. The “in crystallo” selection experiments lead to the discovery of one potentially universal RNA crystal engineering tactic, and it is reasonable to expect that additional principles will be suggested by the structures of other mutant RNAs from future “in crystallo” selection experiments. While this study failed to improve diffraction quality, it demonstrated that “in crystallo” selection can be applied to RNA, and it opened a new approach to promoting RNA crystallization.

Another under-exploited area is the enhancement of the RNA structure stability by introducing X bonds between halogen atoms in bases and the backbone oxygen atoms. These interactions have been better characterized in DNA [108]. Bromine atoms are routinely introduced in synthetic RNAs to obtain experimental phases for structure determination. The position of the halogenated base within an RNA fragment can shift the equilibrium between duplex formation and hairpin formation, so several constructs may have to be tested [109]. Constructs could be prescreened for their conformation by native gel electrophoresis.

Another rapidly emerging structure determination technique of possible relevance to RNA crystallography is microcrystal electron diffraction (MicroED). These experiments are conducted with a transmission electron microscope in diffraction mode. The advantage of this method is that high-resolution diffraction data can often be obtained from nanocrystals (at least one dimension smaller than 100 nm) that are a billionth of the volume considered suitable for X-ray diffraction studies [110]. The disadvantages are the very limited access to instruments that can rotate the sample during data collection. The early successes with peptides and small proteins suggest that it might work well with smaller RNAs. Further technological advancements may be required for success with crystals of larger RNAs.

## 9. Future Relevance of Engineering Crystal Packing in RNA Structures

With the remarkable advancement in structural prediction algorithms and Cryo-EM, one may ask if there is still relevance in designing crystal contacts for nucleic acid structure determination. While the accuracy of AlphaFold2 is remarkable for predicting protein structures [111,112], it may still take some time for nucleic acids structure prediction due to the sparsity of structures available to train a neural network model for RNA structure prediction. Suppose the deep learning approach eventually succeeds at making accurate predictions of nucleic acid structure. In that case, can the deep learning models be harnessed to assist in engineering crystal contacts? Alternatively, other computational approaches from the protein re-design field, like the dead-end elimination theorem [113], could be used to perform the above-mentioned bulge walking in-silico in the presence of all of the surrounding symmetry mates. Such a computational approach to crystal lattice engineering should now be within reach thanks to the increased computational power available from GPUs. For instance, molecular docking of large libraries of small molecules against ensembles of protein structures is now practical thanks to GPUs. Ultimately, predicted structures will never replace the need for experimentally-determined structures designed with biological functions in mind.

With the recent “resolution revolution” in Cryo-EM, Cryo-EM has yielded several low-resolution structures of single-stranded RNAs as small as 40 kDa, suggesting that Cryo-EM will generally apply to large and medium-sized RNA structures [114,115]. It remains to be shown if such low-resolution structures are accurate enough to inform the design of constructs for crystallization to obtain high-resolution structures. Cryo-EM has great promise in the study of RNAs that have conformational heterogeneity, especially if the alternate conformations can be clustered in a modest number of conformations. With suitable clustering algorithms, most of the conformations of a small ensemble can be characterized from a single sample. Thus, Cryo-EM can give multiple structures from one sample. If the conformational heterogeneity cannot be parsed into distinct clusters, it can be addressed computationally at the expense of reduced resolution of the Cryo-EM map.

**Figure 2.**
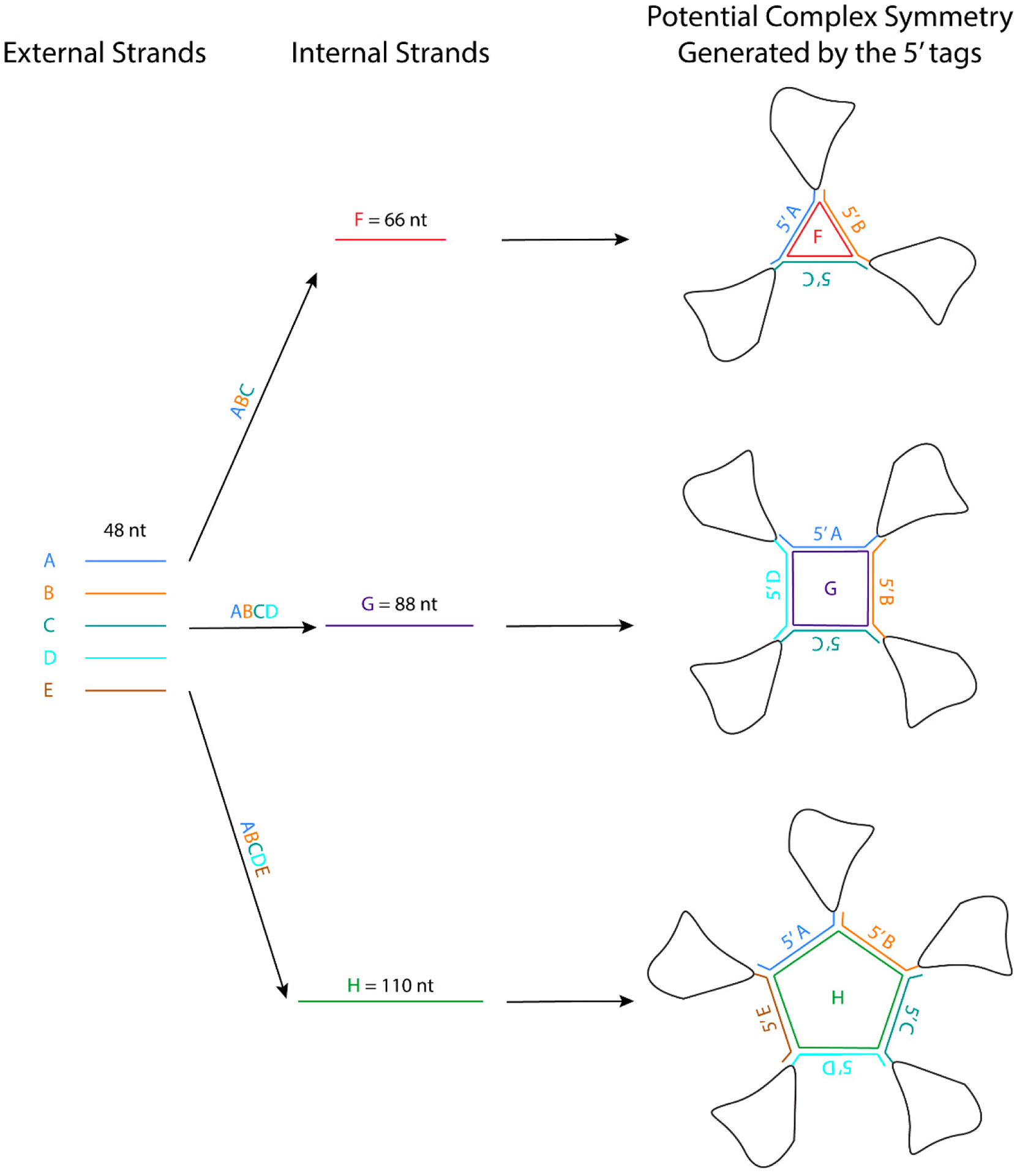
Potential symmetry generation using 3WJ junction forming oligonucleotides (Adapted from Khisamutdinov et al., 2014 [117]). External strands can be cloned into the 5′ end of a complex. Internal strands can be supplied into the sample to induce polygon formation.

The crystal packing strategies described in this review can reduce the flexibility of the interacting regions. Some of these crystal packing modules generate symmetry, which should promote crystallization because proteins with molecular symmetry are known to crystallize more readily than those without molecular symmetry [116]. For example, the kissing loop complex generates two-fold symmetry, the G-quadruplex generates a four-fold symmetry, and the 3WJ junction has been further engineered to form a stable planar triangle, square, and pentagon using oligonucleotides [117]. These polygons are formed using different external 48-nt strands that form the side and one internal strand that base pairs with each external strand like a tape (Figure 2). The affinity of the strands to form the polygon is in the order of ~20 nM, and the formation is highly efficient. An RNA structure can potentially be engineered at the 3′ end of each external strand, allowing the RNA to assemble into a higher-order complex with the polygon in the middle. The addition of these polygons could facilitate Cryo-EM studies by increasing the molecular weight of the RNA and overcoming preferred orientation. If the attached RNAs of interest have consistent orientations with regards to the polygon, they can also provide a rotational symmetry during 3D reconstruction.

## 10. Conclusions

Over 98% of the human genome codes for noncoding RNAs [118]. In vivo genome-wide probing of RNA structures shows the dynamic structural characteristics of mRNA that correlate to cellular physiology [119]. Thus, there are likely many RNA structures with important functions waiting to be discovered. Nucleic acid crystallography will remain a vital structural approach in the years to come. Crystal packing design will continue to be an essential prerequisite for crystallization success and potentially also facilitate Cryo-EM investigations.
